# Artificial Synapse Emulated by Indium Tin Oxide/SiN/TaN Resistive Switching Device for Neuromorphic System

**DOI:** 10.3390/nano13172477

**Published:** 2023-09-01

**Authors:** Dongyeol Ju, Sunghun Kim, Sungjun Kim

**Affiliations:** Division of Electronics and Electrical Engineering, Dongguk University, Seoul 04620, Republic of Korea

**Keywords:** resistive switching, neuromorphic system, synaptic plasticity, Hebbian learning rules, short-term memory

## Abstract

In this paper, we fabricate an ITO/SiN/TaN memristor device and analyze its electrical characteristics for a neuromorphic system. The device structure and chemical properties are investigated using transmission electron microscopy and X-ray photoelectron spectroscopy. Uniform bipolar switching is achieved through DC sweep under a compliance current of 5 mA. Also, the analog reset phenomenon is observed by modulating the reset voltage for long-term memory. Additionally, short-term memory characteristics are obtained by controlling the strength of the pulse response. Finally, bio-inspired synaptic characteristics are emulated using Hebbian learning rules such as spike-rate-dependent plasticity (SRDP) and spike-timing-dependent plasticity (STDP). As a result, we believe that the coexistence of short-term and long-term memories in the ITO/SiN/TaN device can provide flexibility in device design in future neuromorphic applications.

## 1. Introduction

The amount of data we can access is continuously expanding as industrial technology advances. Significant issues arise as a result of the access speed disparity between the CPU resistor and the main memory in von-Neumann computing architecture, which results in a decrease in data processing performance. As a result, the available data hit a bottleneck, limiting its amount [1]. To overcome the bottleneck problem, a novel computing design known as neuromorphic computing architecture is proposed [2]. Neuromorphic computing architecture is based on parallel processing mimicking the human brain, where information proceeds through pre- and post-synapse chemical interactions. The structure based on an artificial neural network (ANN) has advantages in terms of power consumption due to its ability to process large amounts of data in parallel [3,4].

Additionally, charge-based NAND flash memory is about to face limitations in non-volatile storage technology due to its scaling limits [5]. To address the aforementioned issues, different next-generation non-volatile memories such as phase-change memory (PCM) [6], magnetic random-access memory (MRAM) [7], ferroelectric random-access memory (FRAM) [8], and resistive random-access memory (RRAM) [2,9,10,11,12,13] are emerging. RRAM is a viable contender among them due to its high scalability, low-power operation, fast switching speed, long retention time, and high endurance [14,15,16,17,18,19,20,21]. Moreover, due to the simple metal–insulator–metal structure, RRAM has the additional benefits of simple fabrication and mimicking the human brain, where the top electrode metal imitates the pre-synapse and the bottom electrode metal mimics the post-synapse [22]. It is reported that various metal oxides can act as an insulating active layer of RRAM, including TaO_x_ [23], HfO_x_ [24], AlO_x_ [25], ZnO [26], and ZrO_x_ [27]. Under applied bias, these metal oxides form a conducting filament through oxygen-vacancy migration, inducing a change in their resistance state. However, an unstable interface is formed between the electrode and the insulating layer, creating fluctuations in cycle-to-cycle uniformity because of the high reactivity of oxygen ions [28,29]. Recently, studies have focused on nitride-based insulating layers such as AlN [30], SiN [31,32], and WN [33] because of their excellent electrical properties and ionic chemistry, providing a choice to solve the mentioned problems of metal oxides [34,35]. The performance of SiN-based resistive switching devices compared with metal-oxide-based resistive switching devices is summarized in Table 1.

Silicon nitride has been widely studied as a passivation and buffer layer in complementary metal–oxide–semiconductor (CMOS) technology. It is also utilized in flash memory as a charge-trapping layer. Recently, researchers have reported the usage of SiN in RRAM applications, where it is used as a switching layer due to its rich inert defects [36,37,38]. Furthermore, SiN films were reported to have advantages in resistive switching processes such as quick switching speed, extended retention time, high cycle durability, and a large on/off ratio [39,40,41]. Some studies have concentrated on the synaptic applications of SiN-based RRAM devices. Kim et al. reported the co-existence of short-term memory (STM) and long-term memory (LTM) characteristics of Ni/SiN_x_/SiO_2_/Si devices [42]. Choi et al. described the potentiation and depression properties of Ni/SiN_x_/Si devices [43]. Rahmani et al. observed spike-timing-dependent plasticity behavior in Ni/SiN/BN/Si devices [44].

In this work, we investigate more kinds of long- and short-term characteristics of ITO/SiN/TaN. The top electrode ITO is chosen because of its high inert resistance, which can prevent a hard breakdown of the device [45,46]. The device exhibits affirmative characteristics as a memory device, showing good endurance and retention characteristics over 1000 cycles and 10^4^ s, respectively. Additionally, various pulse tests are conducted on the device to obtain short-term characteristics such as paired-pulse facilitation (PPF) [47], paired-pulse depression (PPD) [48], and electric excitatory postsynaptic current (EPSC) [49,50] by altering the compliance current, pulse interval, and pulse width. Finally, Hebbian rules are tested to seek neuromorphic applications [51]. The tests involve potentiation and depression under identical and incremental pulse schemes, spike-rate-dependent plasticity (SRDP), and spike-timing-dependent plasticity (STDP), proving its future applications as a memory device [52,53,54].

## 2. Materials and Methods

The ITO/SiN/TaN device was prepared by performing the following steps. A 100 nm-thick TaN bottom electrode was deposited on a SiO_2_/Si wafer through DC sputtering using a Ta target (99.99% purity) under a power of 65 W. With a deposition pressure of 5 mTorr, the sputtering gas was a mixture of Ar (19 sccm) and N_2_ (1 sccm). A 6 nm-thick insulating SiN film was deposited through plasma-enhanced chemical vapor deposition (PECVD) (Oxford Instruments, Tubney Woods, Abingdon, UK) at a temperature of 250 °C and an RF power of 100 W. The reactive gas consisted of SiH_4_ (5%), He (95%), NH_3_ (9 sccm), and N2 (450 sccm). Then, using photolithography, square patterns of 100 µm × 100 µm were made. Finally, a 110 nm thick Indium Tin Oxide (ITO) top electrode was achieved through a lift-off process in acetone after depositing ITO through RF sputter using a commercial ITO target (99.99% purity) with an RF power of 80 W. The gas pressure was 3 mTorr with Ar of 8 sccm. To determine the DC and pulse characteristics of the device, the electrical properties were characterized using a Keithley 4200-SCS semiconductor parameter analyzer (Keithley Instruments, Cleveland, OH, USA) and a 4225-PMU pulse measuring unit (Keithley Instruments, Cleveland, OH, USA). The top electrode (ITO) was biased, while the bottom electrode (TaN) was grounded. X-ray photoelectron (XPS) analysis and transmission electron microscopy (TEM) (Oxford Instruments, Tubney Woods, Abingdon, UK) were used to examine the depositions and chemical properties of the device.

## 3. Results and Discussion

**Figure 1:**
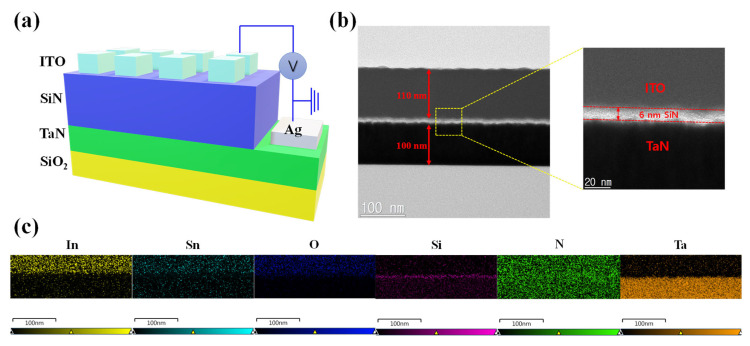
(**a**) Schematic illustration of the ITO/SiN/TaN memristive device structure. (**b**) Typical cross-sectional TEM image of ITO/SiN/TaN structure. (**c**) EDS color mapping of In, Sn, O, Si, N, and Ta.

The schematic illustration of the fabricated ITO/SiN/TaN device is shown in Figure 1a. In Figure 1b, the cross-sectional image of an ITO/SiN/TaN device is investigated through transmission electron microscopy (TEM). It is observed that the film thickness of PECVD-deposited amorphous SiN is about 6 nm. It is noted that the surface of the SiN film is not flat. The surface roughness could produce the electric field that affects the resistive switching [55,56]. The uneven surface can cause the variation in switching parameters even though the enhancement of the electric field can reduce the switching voltage [57].

**Figure 2:**
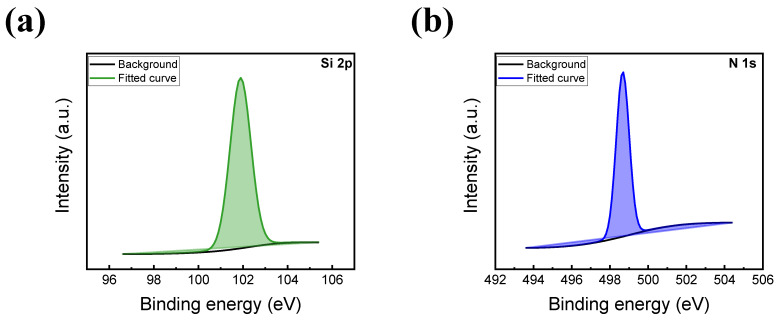
XPS spectra of (**a**) Si 2p and (**b**) N 1s.

The top and bottom electrode thicknesses are about 110 nm and 100 nm, respectively. Figure 1c shows the energy dispersion X-ray spectroscopy mapping of In, Sn, O, Si, N, and Ta elements in the ITO/SiN/TaN device. The spatial distribution of each element in the ITO/SiN/TaN device is observed. It is shown that the nitrogen overlaps the area of ITO, which can be inferred as the formation of an ITON layer. To further investigate the chemical compositions of a 6 nm-thick insulating layer, an XPS in-depth profile mode is conducted. The XPS spectra of the SiN film at the etch time of 6 s are shown in Figure 2. Figure 2a shows the Si 2p spectra, where its peak is located at a binding energy of around 101.97 eV for the Si-N bond [32]. Furthermore, the N 1s spectra are illustrated in Figure 2b with a peak at 498.32 eV, corresponding to the Si-N bond. Nitrogen diffuses during the reactive sputtering process of ITO deposition on a silicon nitride layer.

**Figure 3:**
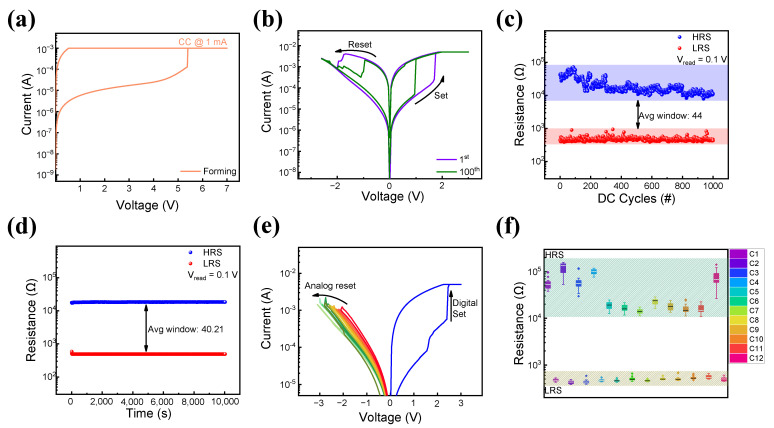
(**a**) Initial breakdown process of ITO/SiN/TaN device. (**b**) I-V curves of the 1st and 100th DC sweep cycles. (**c**) DC endurance performance over 10^3^ cycles. (**d**) Retention characteristics of HRS and LRS over 10^4^ s. (**e**) Analog reset process of the device. (**f**) Cell-to-cell uniformity over 10 cells.

Next, the electrical characteristics of the ITO/SiN/TaN device are investigated. As shown in Figure 3a, the electrical soft breakdown process [52,53] under a voltage of 5.5 V and a compliance current of 1 mA is needed to switch the device from its initial resistance state to a low-resistance state (LRS). The strong bias applied to the top electrode gathers the initial randomly scattered defects in the SiN thin film and forms an initial conducting filament, increasing the flow of current. The set and reset processes occur under voltage biases of about 3 V and –3 V, with a compliance current of 5 mA. The first and 100th I–V cycles are shown in Figure 3b. The high-resistance state (HRS) and LRS are repeatedly switched without severe breakdown. To further observe the cycle-to-cycle variance of the device, a DC sweep over 10^3^ cycles is performed as shown in Figure 3c. Each resistance state is distinguishable during the cycles, obtaining an average on/off ratio of 43.98. Figure 3d shows the retention characteristics of the device in the LRS and HRS at a read voltage of 0.1 V. Each LRS and HRS maintains their resistance level over 10^4^ s, displaying promising non-volatile memory properties [49]. Furthermore, analog reset, which is critical for neuromorphic computing applications for multi-level states [58,59], is illustrated in Figure 3e. The set process occurs in a single digital set process at 3 V and a sequential reset process occurs by modulating the reset voltage from –2 V to –3 V. To confirm the cell-to-cell uniformity of ITO/SiN/TaN, 10 randomly selected cells are tested under the same voltage bias of 3 V and –3 V over 20 cycles. As shown in Figure 3f, a significant difference in HRS is observed in all 10 cells, showing its cell-to-cell uniformity.

**Figure 4:**
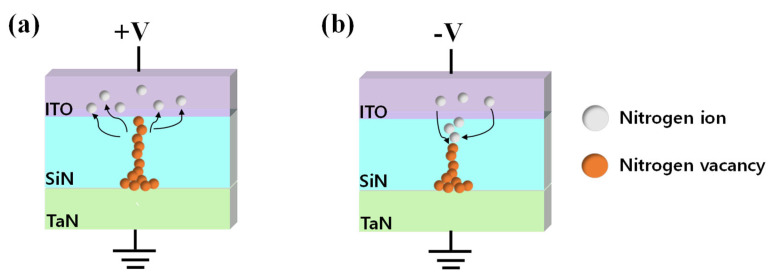
Conduction mechanism of ITO/SiN/TaN device. (**a**) Set process. (**b**) Reset process.

Figure 4 illustrates a conduction mechanism based on the migration of nitride vacancies based on the I–V characteristics of Figure 3. When the top electrode is biased positively, nitride ions (N^3−^) move toward the top electrode ITO, forming a nitride-rich ITON layer that works as a nitride reservoir. The remaining nitride vacancies (silicon dangling bond) aggregate and form a conducting filament toward the top electrode. Under continuous voltage bias, the filament tip contacts the top electrode and the device, and a large amount of current flows through conducting filament. Thus, the device switches from an HRS to an LRS, which is an “on” state (Figure 4a) [60,61]. On the other hand, when a negative voltage is applied to the top electrode, nitride ions that have gathered in the ITON region are expelled to their original location, recombining with the nitrogen-vacancy and rupturing the conducting path. As a result, the current level of the device decreases, switching it from an LRS to an HRS, and the device is turned “off” (Figure 4b) [62].

**Figure 5:**
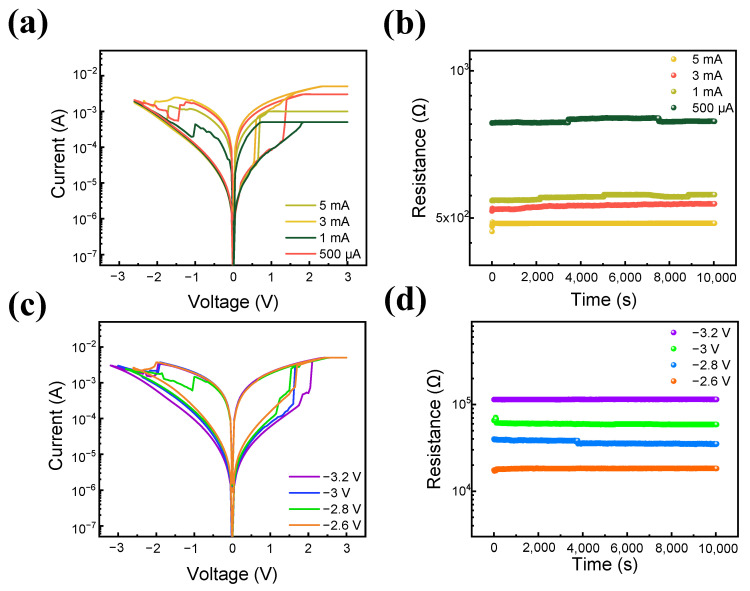
(**a**) MLC characteristics under different compliance currents. (**b**) Retention characteristics of MLC under different compliance currents. (**c**) MLC characteristics under different reset voltages. (**d**) Retention characteristics of MLC under different reset voltages.

Multi-level cell (MLC) characteristics are important factors in memory devices due to their practical applications in high-density memory and neuromorphic devices [63,64,65,66]. In a single-level cell (SLC), for example, one cell retains two bits, meaning that the cell only remains in two types of resistance states: LRS and HRS [67]. On the other hand, MLCs can display multiple resistance states, increasing the storage density. Two controllable approaches exist to embody MLCs; the first is by controlling the compliance current in the set operation [68]. During the set operation, defects accumulate, forming a conducting filament. In this process, compliance current is applied to avoid the hard breakdown of the device by limiting the size of the conducting filament and restricting the current flow. Thus, the current flow can be optimized by the applied compliance current, resulting in multiple LRS states while the HRS level remains the same. As shown in Figure 5a, while the off current remains at the same level in all compliance current statuses, the on current can be varied. The second way to obtain MLC is by changing the reset voltage [69]. In the reset process, nitride ions that migrate into the ITON layer repel back to their original space. This can be noted as the amount of repelled ions can be controlled by the strength of the reset voltage applied to the top electrode, controlling the rupture of the conducting filament. Thus, when a strong reset voltage is applied, the conducting path will be more ruptured, resulting in a multi-HRS state with the same LRS. Figure 5c shows the I-V of the ITO/SiN/TaN device with different reset voltages applied from −2.6 V to −3.2 V. As shown, four different acquired I-Vs have the same LRS but differing HRS. To determine the ability of the device to maintain its multi-level states as a non-volatile memory, retention characteristics are tested and read at 0.1 V. The result is illustrated in Figure 5b,d, where the states remain in their resistance state over 10^4^ s without significant variance.

**Figure 6:**
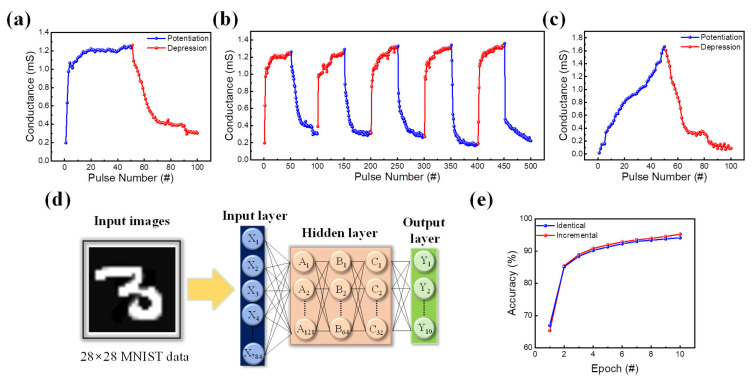
(**a**) Potentiation and depression. (**b**) 5-cycle potentiation and depression. (**c**) Result of the incremental pulse scheme applied to the ITO/SiN/TaN device. (**d**) Deep neural network simulation framework for MNIST pattern recognition. (**e**) The pattern recognition accuracy of a synaptic device over ten consecutive epochs.

In the human brain, various information processes occur to realize memory. This information processing is frequently influenced by synaptic plasticity, which is a method for modulating synaptic weight between the post- and pre-synapse. The synaptic weight is updated by the interval time of electrical input between the pre- and post-synapse [70,71]. To mimic a biological synapse, AC pulse analyses are usually used. One way to perform neuromorphic applications on an electronic synaptic device is through potentiation and depression. Long-term potentiation (LTP) and long-term depression (LTD) characteristics are found in ITO/SiN/TaN devices by applying pulses of 50 identical set pulses and 50 identical reset pulses. The pulse train is composed of a pulse width and an interval of 1 μs. The pulse voltage is 2.5 V and −2.5 V for each set, and the reset pulse train with data reads at a read voltage of 0.1 V. Figure 6a shows an increase and decrease in conductance states. A five-cycle pulse cycle of LTP and LTD is demonstrated by the consecutive set and consecutive reset pulses in Figure 6b. However, when developing neuromorphic computing systems, the linearity and asymmetry of LTP and LTD are critical considerations [72]. To regulate the non-linearity of the LTP process, we use an incremental pulse train with a voltage ranging from 1.8 V to 2.5 V on the device. The result of incremental pulse application is shown in Figure 6c, showing the improved linearity of conductance change. To further seek the application of ITO/SiN/TaN devices in neuromorphic computing applications, the pattern recognition system (PRS) based on the Modified National Institute of Standards and Technology database (MNIST) is tested using Python and Google Colab. The PRS is divided into three categories, as indicated in Figure 6d: the input, output, and hidden neurons. The conductance graph of LTP and LTD is translated into a 28 × 28 MNIST handwritten image where the pixel change follows the change in conductance. Then, the numbers are inserted into PRS, where neurons in each layer calculate the correctness of the handwritten image. Figure 6e shows the calculated accuracy in which the gradual and linear incremental pulse scheme has a better accuracy of 97.22%.

**Figure 7:**
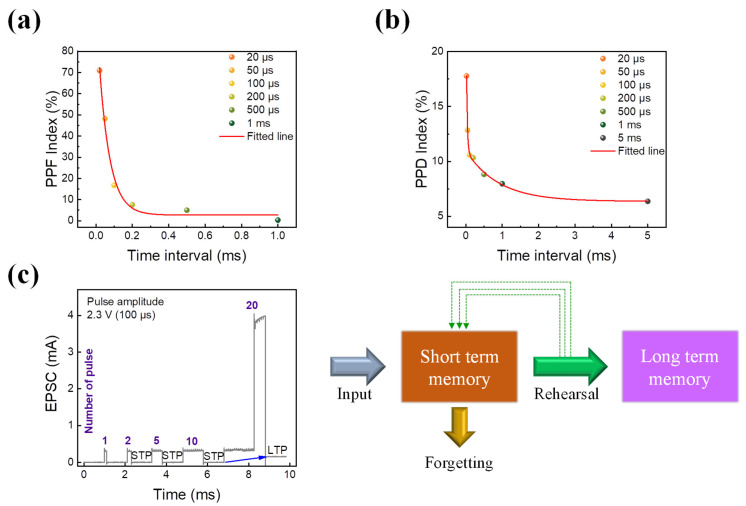
(**a**) PPF measurement. (**b**) PPD measurement. (**c**) The transition of STM to LTM of ITO/SiN/TaN device with the change in EPSC.

In addition to long-term memories, the human brain has short-term memories that we swiftly forget. Thus, when emulating the human brain, a cohabitation of short- and long-term actions may be incorporated as an added strength [42,73]. PPF is a standard method to seek short-term memory behavior by applying two identical pulse trains with different pulse intervals [74]. Figure 7a shows the PPF index that is defined as ((I_2_ − I_1_)/I_1_) × 100, where I_1_ is the current achieved by the first pulse train and I_2_ is the current achieved by the next pulse train. When the pulse interval is short, the second pulse is affected by the first, resulting in a larger current. When the gap is sufficiently long (1 ms), the device forgets the first pulse, resulting in no change in current by the second pulse. In addition, PPD is used to confirm the short-term properties of an ITO/SiN/TaN device [48]. The measurement is conducted by varying pulse intervals between two consecutive pulse trains. The term PPD index is also defined as ((I_2_ − I_1_)/I_1_) × 100. As shown in Figure 7b, the current responds by the second pulse decreasing as the interval increases, well mimicking short-term memory. Furthermore, by rehearsing the applied simulation, short-term memory can be convolved into long-term memory at the biological synapse [75]. Thus, a pulse test with varying numbers of pulses is applied to calculate the change in EPSC. The pulse amplitude and width are 2.3 V and 100 s, respectively, while the pulse number ranges from 1 to 20. When the number of pulses is small, as shown in Figure 7c, the current abruptly climbs and falls, which can be implemented as STM characteristics. However, when 20 identical pulses are administered, the read current increases, indicating LTM properties, where STM is changed into LTM through event rehearsal.

**Figure 8:**
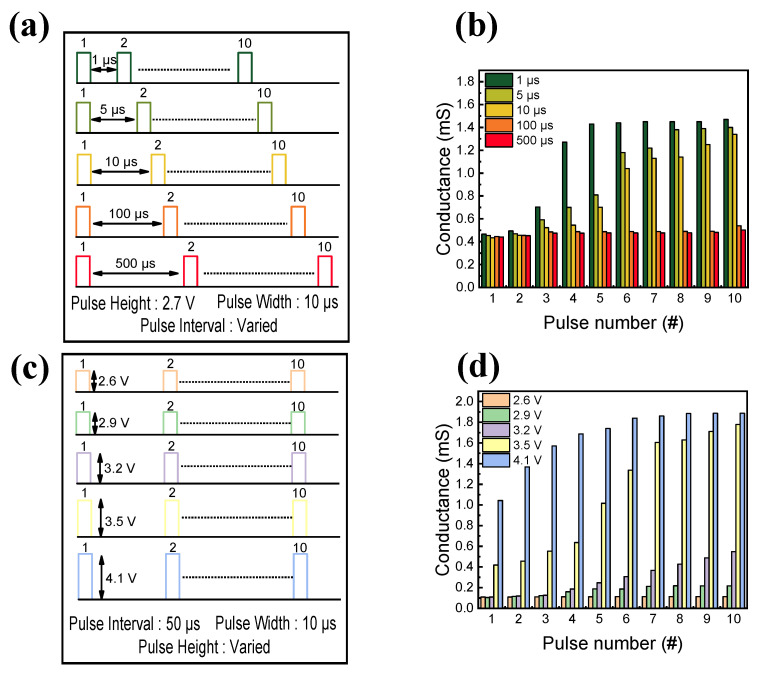
(**a**) Schematic illustration of the designed pulse scheme for the demonstration of the SRDP function. (**b**) Conductance response of the SRDP function. (**c**) Schematic illustration of the designed pulse scheme for the demonstration of spike-amplitude-dependent synaptic behavior. (**d**) The conductance response of spike-amplitude-dependent synaptic function.

Hebbian principles are frequently used to assess a device’s ability to simulate pre- and post-synaptic neurons and their functions. One of those learning rules is SRDP, which tests the synaptic response under different frequency simulations [76]. To demonstrate the SRDP function, sets of 10 identical pulse trains with different pulse intervals are prepared. The amplitude and width of the pulse are 2.7 V and 10 s, respectively. To obtain SRDP, the interval is varied from 1 μs to 500 μs, as shown in Figure 8a. Figure 8b shows a desirable relationship between pulse interval and conductance. A quick escalation of conductance is found for short pulse intervals, whereas the conductance update is smaller for larger pulse intervals. Furthermore, synaptic functions by altering pulse amplitude are also tested. As depicted in Figure 8c, a fixed pulse interval and width of 50 μs and 10 μs are applied to observe the conductance change. The varied amplitude of the pulse ranges from 2.6 V to 4.1 V. Figure 8d shows the linear relationship between pulse amplitude and conductance. Rapid conductance changes are observed for large pulse amplitudes, similar to the SRDP result. In the event of a small pulse amplitude, however, no significant variation is observed.

**Figure 9:**
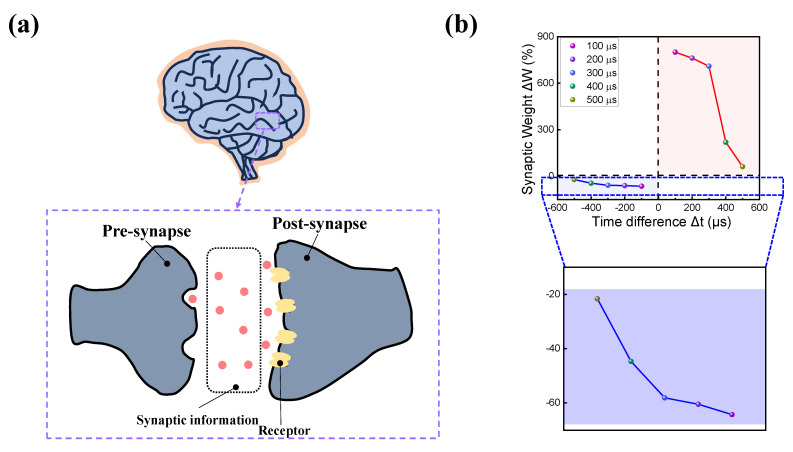
(**a**) Schematic illustration of the biological brain’s pre- and post-synapse. (**b**) Synaptic weight as a function of the time difference between two pulse trains.

In addition to mimicking the function of the biological synapse, duplicating its physical form can bring value to the realization of an efficient neuromorphic system [72]. As shown in Figure 9a, the structure of a biological synapse is located between the post- and pre-synapse. The synaptic information from the pre-synapse migrates toward the post-synapse receptor, which passes the information to the post-synapse. RRAM, for example, has a simple two-terminal structure that can easily mimic a biological synapse, where the top electrode imitates the pre-synapse, and the bottom electrode imitates the post-synapse. The synaptic information can be changed by the formation of a conducting filament. On the other hand, the memristor conductance is regarded as synaptic weight. Another Hebbian rule known as STDP is investigated to efficiently replicate the biological structure and synaptic weight change of the top and bottom electrodes [77,78]. In the STDP, pulse trains of the same components are fired at both the pre-and post-synapse with a time difference. The time difference between synapses decides whether the device experiences LTP or LTD. The time difference (Δt) between post- and pre-synapses is defined as Equation (1):

Δt = t_post_ − t_pre_ (1)

where t_post_ and t_pre_ are the times when pulses are applied to the pre- and post-synapses, respectively. When the pre-synapse exceeds the post-synapse (Δt > 0), effective positive set pulse trains are applied, resulting in a decrease in resistance, and LTP occurs. On the other hand, when the post-synapse exceeds the pre-synapse (Δt < 0), effective negative reset pulse trains are applied, increasing resistance, and LTD occurs. The result of applying a pair of pulse trains is illustrated in Figure 9b, where the synaptic weight (Δ*W*) is defined as Equation (2):

(2) $$\Delta W=\frac{G_{f}-G_{i}}{G_{i}}\times 100\left(\%\right)$$

where *G_f_* and *G_i_* are the conductance values of the device after and before applying a pair of pulses, respectively. As revealed in the results, the synaptic weight is greatest when the time gap between the pre- and post-synapse is smallest and the synaptic weight decreases as the time difference is large, replicating the STDP behavior.

## 4. Conclusions

In summary, we fabricated a SiN-based memristor to investigate the resistive switching characteristics and synaptic functions. The nitride-based resistive switching shows uniform resistive switching for 10^3^ s with a large on/off window. The MLC characteristics are obtained by varying the reset voltage and compliance current, which enlarge the data storage of the device. The coexistence of STM and LTM is found during the pulse scheme, whereby PPF, PPD, and EPSC are demonstrated to emulate bio-inspired nerve systems. Finally, we demonstrate that the ITO/SiN/TaN device could mimic the human brain’s Hebbian rules.

## Figures and Tables

**Table 1:** Performance of nitride-based devices compared with oxide-based devices in previous reports.

No	Structure	Endurance	On/Off Ratio	Retention	Ref.
1	ITO/WO_x_/TiN	<10	NA	NA	[2]
2	ITO/WO_x_/TaO_x_/TiN	>10^2^	>10	>10^4^	[2]
3	Ag/ZnO/TiN	<936	<10	NA	[18]
4	TiN/SiOx/ITO	>2 × 10^2^	NA	>10^4^	[19]
5	ITO/SiN/TaN	>10^3^	>43	>10^4^	This work

## Data Availability

Not applicable.

## References

[1] Sangwan V.K., Hersam M.C. (2020). Neuromorphic nanoelectronic materials. Nat. Nanotechnol..

[2] Hong X., Loy D., Dananjaya P., Tan F., Ng C., Lew W.J. (2018). Oxide-based RRAM materials for neuromorphic computing. Mater. Sci..

[3] Borghetti J., Snider G.S., Kuekes P.J., Yang J.J., Stewart D.R. (2010). ‘Memristive’ switches enable ‘stateful’ logic operations via material implication. Nature.

[4] Gao S., Zeng F., Wang M., Wang G., Song C., Pan F. (2015). Implementation of complete Boolean logic functions in single complementary resistive switch. Sci. Rep..

[5] Theis T.N., Wong H.S.P. (2017). The end of moore’s law: A new beginning for information technology. Comput. Sci. Eng..

[6] Fong S.W., Neumann C.M., Wong H.S.P. (2017). Phase-change memory—Towards a storage-class memory. IEEE Trans. Electron. Devices.

[7] Khvalkovskiy A.V., Apalkov D., Watts S., Chepulskii R., Beach R.S., Ong A., Drinskill-Smith X.A., Butler W.H., Visscher P.B. (2013). Basic principles of STT-MRAM cell operation in memory arrays. J. Phys. D Appl. Phys..

[8] Hiroshi I.J. (2012). Ferroelectric random access memories. Nanosci. Nanotechnol..

[9] Sawa A. (2008). Resistive switching in transition metal oxides. Mater. Today.

[10] Park J., Park H., Chung D., Kim S. (2022). Dynamic and Static Switching in ITO/SnOx/ITO and Its Synaptic Application. Int. J. Mol. Sci..

[11] Jhang W.C., Hsu C.C. (2022). Dual-Function Device Fabricated Using One Single SiO2 Resistive Switching Layer. IEEE Electron. Device Lett..

[12] Park M., Jeon B., Park J., Kim S. (2022). Memristors with Nociceptor Characteristics Using Threshold Switching of Pt/HfO2/TaOx/TaN Devices. Nanomaterials.

[13] Kim D., Shin J., Kim S. (2021). Resistive Switching Characteristics of ZnO-Based RRAM on Silicon Substrate. Metals.

[14] Jeong D.S., Thomas R., Katiyar R.S., Scott J.F., Kohlstedt H., Petraru A., Hwang C.S. (2012). Emerging memories: Resistive switching mechanisms and current status. Rep. Prog. Phys..

[15] Waser R., Aono M. (2007). Nanoionics-based resistive switching memories. Nat. Mater..

[16] Ventra M.D., Pershin Y.V. (2011). Memory materials: A unifying description. Mater. Today.

[17] Pan F., Gao S., Chen C., Song C., Zeng F. (2014). Recent progress in resistive random access memories: Materials, switching mechanisms, and performance. Mater. Sci. Eng. R-Rep..

[18] Chand U., Huang C.Y., Jieng J.H., Jang W.Y., Lin C.H., Tseng T.Y. (2015). Suppression of endurance degradation by utilizing oxygen plasma treatment in HfO2 resistive switching memory. Appl. Phys. Lett..

[19] Choi J., Le Q.V., Hong K., Moon C.W., Han J.S., Kwon K.C., Cha P.R., Kwon Y., Kim S.Y., Jang H.W. (2017). Enhanced endurance organolead halide perovskite resistive switching memories operable under an extremely low bending radius. ACS Appl. Mater. Interfaces.

[20] Yu M.J., Son K.R., Khot A.C., Kang D.Y., Sung J.H., Jang I.G., Dange Y.D., Dongale T.D., Kim T.G. (2021). Three Musketeers: Demonstration of multilevel memory, selector, and synaptic behaviors from an Ag-GeTe based chalcogenide material. J. Mater. Res. Technol-JMRT.

[21] Zhu Y.B., Zheng K., Wu X., Ang L.K. (2017). Enhanced stability of filament-type resistive switching by interface engineering. Sci. Rep..

[22] Roy S., Niu G., Wang Q., Wang Y., Zhang Y., Wu H., Zhai S., Shi P., Song S., Song Z. (2020). Toward a Reliable Synaptic Simulation Using Al-Doped HfO2 RRAM. ACS Appl. Mater. Interfaces.

[23] Prakash A., Jana D., Maikap S. (2013). TaO x -based resistive switching memories: Prospective and challenges. Nanoscale Res. Lett..

[24] Tan T., Du Y., Cao A., Sun Y., Zhang H., Zha G. (2018). Resistive switching of the HfOx/HfO 2 bilayer heterostructure and its transmission characteristics as a synapse. RSC Adv..

[25] Zhang X., Xu L., Zhang H., Liu J., Tan D., Chen L., Ma Z., Li W. (2020). Effect of Joule Heating on Resistive Switching Characteristic in AlOx Cells Made by Thermal Oxidation Formation. Nanoscale Res. Lett..

[26] Oh I., Pyo J., Kim S. (2022). Resistive Switching and Synaptic Characteristics in ZnO/TaON-Based RRAM for Neuromorphic System. Nanomaterials.

[27] Wang S.Y., Tsai C.H., Lee D.Y., Lin C.Y., Lin C.C., Tseng T.Y. (2011). Improved resistive switching properties of Ti/ZrO2/Pt memory devices for RRAM application. Microelectron. Eng..

[28] Lin S., Wu C., Chang T., Lien C., Yang C., Chen W., Lin C., Huang W., Tan Y., Wu P. (2021). Improving Performance by Inserting an Indium Oxide Layer as an Oxygen Ion Storage Layer in HfO_2_-Based Resistive Random Access Memory. IEEE Trans. Electron. Devices.

[29] Chen P.H., Su Y.T., Chang F.C. (2019). Stabilizing Resistive Switching Characteristics by Inserting Indium-Tin-Oxide Layer as Oxygen Ion Reservoir in HfO2-Based Resistive Random Access Memory. IEEE Trans. Electron. Devices.

[30] Yang S., Park J., Cho Y., Lee Y., Kim S. (2022). Enhanced Resistive Switching and Synaptic Characteristics of ALD Deposited AlN-Based RRAM by Positive Soft Breakdown Process. Int. J. Mol. Sci..

[31] Sun B., Han X., Xu R., Qian K. (2020). Uncovering the Indium Filament Formation and Dissolution in Transparent ITO/SiNx/ITO Resistive Random Access Memory. ACS Appl. Electron. Mater..

[32] Park J., Lee S., Lee K., Kim S. (2021). Conductance quantization behavior in pt/sin/tan rram device for multilevel cell. Metals.

[33] Hong S.M., Kim H.D., An H.M., Kim T.G. (2013). Resistive switching phenomena of tungsten nitride thin films with excellent CMOS compatibility. Mater. Res. Bull..

[34] Yang M., Wang H., Ma X., Gao H., Wang B. (2017). Effect of nitrogen-accommodation ability of electrodes in SiNx-based resistive switching devices. Appl. Phys. Lett..

[35] Kim H.D., An H.M., Lee E.B., Kim T.G. (2011). Stable bipolar resistive switching characteristics and resistive switching mechanisms observed in aluminum nitride-based ReRAM devices. IEEE Trans. Electron. Devices.

[36] Yoom S.O., Morrow E.S. (2019). Evidence of preserved audience design with aging in interactive conversation. Psychol. Aging.

[37] Jiang X., Ma Z., Xu J., Chen K., Xu L., Li W., Huang X., Feng D. (2015). a-SiNx:H-based ultra-low power resistive random access memory with tunable Si dangling bond conduction paths. Sci. Rep..

[38] Vasileiadis N., Karakolis P., Mandylas P., Ioannou-Sougleridis V., Normand P., Perego M., Komninou P., Ntinas V., Fyrigos I.A., Karafyllidis I. (2021). Understanding the role of defects in silicon nitride-based resistive switching memories through oxygen doping. IEEE Trans. Nanotechnol..

[39] Xia G., Ma Z., Jiang X., Yang H., Xu J., Xu L., Li W., Chen K., Feng D.D.J. (2012). Direct observation of resistive switching memories behavior from nc-Si embedded in SiO2 at room temperature. Non-Cryst. Solids.

[40] Chang Y.F., Fowler B., Chen Y.C., Chen Y.T., Wang Y., Xue F., Zhou F., Lee J.C. (2014). Intrinsic SiOx-based unipolar resistive switching memory. II. Thermal effects on charge transport and characterization of multilevel programing. J. Appl. Phys..

[41] Kim H.D., An H.M., Hong S.M., Kim T.G. (2012). Unipolar resistive switching phenomena in fully transparent SiN-based memory cells. Semicond. Sci. Technol..

[42] Choi J., Kim S. (2020). Coexistence of Long-Term Memory and Short-Term Memory in an SiNx-Based Memristor. Phys. Status Solidi-Rapid Res. Lett..

[43] Kim B., Choi H.S., Kim Y. (2020). A study of conductance update method for Ni/SiNx/Si analog synaptic device. Solid-State Electron..

[44] Rahmani M.K., Kim M.H., Hussain F., Abbas Y., Ismail M., Hong K., Mahata C., Choi C., Park B.G., Kim S. (2020). Memristive and synaptic characteristics of nitride-based heterostructures on si substrate. Nanomaterials.

[45] Ye C., Wu J.J., Pan C.H., Tsai T.M., Chang K.C., Wu H., Deng N., Qian H. (2017). Boosting the performance of resistive switching memory with a transparent ITO electrode using supercritical fluid nitridation. RCS Adv..

[46] Ye C., Zhan C., Tsai T.M., Chang K.C., Chen M.C., Chang T.C., Deng T., Wang H. (2014). Low-power bipolar resistive switching TiN/HfO2/ITO memory with self-compliance current phenomenon. Appl. Phys. Express.

[47] Lin C.Y., Chen J., Chen P.H., Chang T.C., Wu Y., Eshraghian J.K., Moon J., Yoo S., Wang Y.H., Chen W.C. (2020). Adaptive synaptic memory via lithium ion modulation in RRAM devices. Small.

[48] Zhou L., Mao J.Y., Yang J., Zhang S., Zhou Y., Liao Q., Zeng Y., Shan H., Xu Z., Fu J. (2018). Biological spiking synapse constructed from solution processed bimetal core–shell nanoparticle based composites. Small.

[49] Lee Y., Park J., Chung D., Lee K., Kim S. (2022). Multi-level cells and quantized conductance characteristics of Al2O3-based RRAM device for neuromorphic system. Nanoscale Res. Lett..

[50] Lian X., Shen X., Fu J., Gao Z., Wan X., Liu X., Hu E., Xu J., Tong Y.Y. (2020). Electrical properties and biological synaptic simulation of Ag/MXene/SiO2/Pt RRAM devices. Electronics.

[51] Sejnowski T.J., Tesauro G. (1989). The Hebb rule for synaptic plasticity: Algorithms and implementations. Neural Models Plast..

[52] Park J., Kwak M., Moon K., Woo J., Lee D., Hwang H. (2016). TiO x-based RRAM synapse with 64-levels of conductance and symmetric conductance change by adopting a hybrid pulse scheme for neuromorphic computing. IEEE Electron. Device Lett..

[53] Kim D., Lee H.J., Yang T.J., Choi W.S., Kim C., Choi S.J., Bae J.H., Kim D.M., Kim S., Kim D.H. (2022). Effect of post-annealing on barrier modulations in Pd/IGZO/SiO2/p+-Si memristors. Nanomaterials.

[54] Zahoor F., Zulkifli T.Z., Khanday F.A. (2020). Resistive Random Access Memory (RRAM): An Overview of Materials, Switching Mechanism, Performance, Multilevel Cell (mlc) Storage, Modeling, and Applications. Nanoscale Res. Lett..

[55] Ishibe T., Uematsu Y., Naruse N., Mera Y., Nakamura Y. (2020). Impact of metal silicide nanocrystals on the resistance ratio in resistive switching of epitaxial Fe3O4 films on Si substrates. Appl. Phys. Lett..

[56] Yoon J.H., Han J.H., Jung J.S., Jeon W., Kim G.H., Song S.J., Seok J.Y., Yoon K.J., Lee M.H., Hwang C.S. (2013). Highly Improved Uniformity in the Resistive Switching Parameters of TiO2 Thin Films by Inserting Ru Nanodots. Adv. Mater..

[57] Kim S., Jung S., Kim M.H., Kim T.H., Bang S., Cho S., Park B.G. (2017). Nano-cone resistive memory for ultralow power operation. Nanotechnology.

[58] Abbas Y., Jeon Y.R., Sokolov A.S., Kim S., Ku B., Choi C. (2018). Compliance-free, digital SET and analog RESET synaptic characteristics of sub-tantalum oxide based neuromorphic device. Sci. Rep..

[59] Ismail M., Mahata C., Kim S.J. (2022). Forming-free Pt/Al2O3/HfO2/HfAlOx/TiN memristor with controllable multilevel resistive switching and neuromorphic characteristics for artificial synapse. J. Alloys Compd..

[60] Padovani A., Larcher L., Pirrotta O., Vandelli L., Bersuker G. (2015). Microscopic Modeling of HfOx RRAM Operations: From Forming to Switching. IEEE Trans. Electron. Devices.

[61] Raghavan N., Fantini A., Degraeve R., Roussel P.J., Goux L., Govoreanu B., Wouters D.J., Groeseneken G., Jurczak M. (2013). Statistical insight into controlled forming and forming free stacks for HfOx RRAM. Microelectron. Eng..

[62] Zhang Z., Gao B., Fang Z., Wang X., Tang Y., Sohn J., Wong H.S., Wong S.S., Lo G.Q. (2014). All-metal-nitride RRAM devices. IEEE Electron. Device Lett..

[63] Lin J., Wang S., Liu H. (2021). Multi-Level Switching of Al-Doped HfO2 RRAM with a Single Voltage Amplitude Set Pulse. Electronics.

[64] Wu J., Ye C., Zhang J., Deng T., He P., Wang H. (2016). Multilevel characteristics for bipolar resistive random access memory based on hafnium doped SiO2 switching layer. Mater. Sci. Semicond. Process.

[65] Gentili P.L. (2021). Establishing a New Link between Fuzzy Logic, Neuroscience, and Quantum Mechanics through Bayesian Probability: Perspectives in Artificial Intelligence and Unconventional Computing. Molecules.

[66] Chreistensen D.V., Dittmann R., Barranco B.L., Sebastian A., Gallo M.L., Redaelli A., Slesazeck S., Mikolajick T., Spiga S., Menzel S. (2022). 2022 roadmap on neuromorphic computing and engineering. Neuromorphic Comput. Eng..

[67] Hong S., Shin D. (2010). International Workshop on Storage Network Architecture and Parallel I/Os.

[68] Sedghi N., Li H., Brunell I.F., Dawson K., Potter R.J., Guo Y., Gibbon J.T., Dhanak V.R., Zhang W.D., Zhang J.F. (2017). The role of nitrogen doping in ALD Ta2O5 and its influence on multilevel cell switching in RRAM. Appl. Phys. Lett..

[69] Prakash A., Hwang H. (2019). Multilevel cell storage and resistance variability in resistive random access memory. Phys. Sci. Rev..

[70] Malenka R.C. (1994). Synaptic plasticity in the hippocampus: LTP and LTD. Cell.

[71] Malenka R.C. (1995). Review: LTP and LTD: Dynamic and interactive processes of synaptic plasticity. Neuroscientist.

[72] Ismail M., Abbas H., Sokolov A., Mahata C., Choi C., Kim S. (2021). Emulating synaptic plasticity and resistive switching characteristics through amorphous Ta2O5 embedded layer for neuromorphic computing. Ceram. Int..

[73] Du C., Cai F., Zidan M.A., Ma W., Lee S.H., Lu W.D. (2017). Reservoir computing using dynamic memristors for temporal information processing. Nat. Commun..

[74] Prakash C., Dixit A. (2022). Multifunctional BiFeO3 Thin Film-Based Memristor Device as an Efficient Synapse: Potential for Beyond von Neumann Computing in Neuromorphic Systems. ACS Appl. Electron. Mater..

[75] McGaugh J.L. (2000). Memory--a century of consolidation. Science.

[76] Sun J., Fu Y., Wan Q.Q.J. (2018). Organic synaptic devices for neuromorphic systems. Phys. D-Appl. Phys..

[77] Zarudnyi K., Mehonic A., Montesi L., Buckwell M., Hudziak S., Kenyon A.J. (2018). Spike-timing dependent plasticity in unipolar silicon oxide RRAM devices. Front Neurosci..

[78] Ju D., Kim J.H., Kim S.J. (2023). Highly uniform resistive switching characteristics of Ti/TaOx/ITO memristor devices for neuromorphic system. J. Alloys Compd..

